# Expression profiling of lymph node cells from deer mice infected with Andes virus

**DOI:** 10.1186/1471-2172-14-18

**Published:** 2013-04-09

**Authors:** Tony Schountz, Timothy I Shaw, Travis C Glenn, Heinz Feldmann, Joseph Prescott

**Affiliations:** 1 Department of Microbiology, Immunology and Pathology, College of Veterinary Medicine, Colorado State University, Fort Collins, CO, USA; 2 Institute of Bioinformatics, University of Georgia, Athens, GA, USA; 3 Department of Environmental Health Sciences, University of Georgia, Athens, GA, USA; 4 Laboratory of Virology, Division of Intramural Research, National Institute of Allergy and Infectious Diseases, National Institutes of Health, Rocky Mountain Laboratories, Hamilton, MT, USA

## Abstract

**Background:**

Deer mice (*Peromyscus maniculatus*) are the principal reservoir hosts of Sin Nombre virus (SNV), the cause of the great majority of hantavirus cardiopulmonary syndrome (HCPS) cases in North America. SNV, like all hantaviruses with their reservoirs, causes persistent infection without pathology in deer mice and appear to elicit a regulatory T cell response. Deer mice are also susceptible to Andes virus (ANDV), which causes the great majority of HCPS cases in South America, but they clear infection by 56 days post infection without signs of disease.

**Results:**

We examined lymph node cell responses of deer mice infected with ANDV to determine expression profiles upon *in vitro* recall challenge with viral antigen. Because the deer mouse genome is currently unannotated, we developed a bioinformatics pipeline to use known lab mouse (*Mus musculus*) cDNAs to predict genes within the deer mouse genome and design primers for quantitative PCR (http://dna.publichealth.uga.edu/BlastPrimer/BlastPrimer.php). Of 94 genes examined, 20 were elevated, the plurality of which were Th2-specific, whereas 12 were downregulated. Other expressed genes represented Th1, regulatory T cells and follicular helper T cells, and B cells, but not Th17 cells, indicating that many cellular phenotypes participate in the host response to Andes virus.

**Conclusions:**

The ability to examine expression levels of nearly any gene from deer mice should allow direct comparison of infection with SNV or ANDV to determine the immunological pathways used for clearance of hantavirus infection in a reservoir host species.

## Background

Deer mice (*Peormyscus maniculatus*) and other peromyscine species are the most abundant mammals in North America and found from the sub-arctic to Mexico [1]. The deer mouse genome has recently been sequenced to 7x depth but it has not yet been annotated, which presents difficulties for leveraging it as a resource. The genus hosts a number of pathogens, including the agents that cause granulocytic ehrlichiae (*Ehrlichia* spp.), Lyme disease (*Borrelia burgdorferi*), babesiosis (*Babesia microti*), cryptosporidia (*Cryptosporidium parvum*), bartenellosis (*Bartonella vinsonii*), cutaneous leishmaniasis in Mexico and Central America (*Leishmania mexicana*), deer tick virus, Powassan virus, and Sin Nombre (SNV) and New York-1 (NYV) hantaviruses (etiologic agents of hantavirus cardiopulmonary syndrome [HCPS]) [2-17]. These diseases cause significant morbidity and mortality in North and Central America each year, and some are important agricultural pathogens. Despite the importance of this genus as reservoirs and vectors, little is known about the immunology of peromyscines.

Hantaviruses are tri-segmented negative-stranded viruses hosted by rodents or insectivores [18,19]. Several pathogenic hantaviruses, which are rodent-borne, cause HCPS in the Americas or hemorrhagic fever with renal syndrome (HFRS) in Eurasia, both of which have prominent immunopathologic components. The two most important HCPS-causing hantaviruses are SNV, hosted by the North American deer mouse, and Andes virus (ANDV), hosted by the South American long-tailed pygmy rice rat (*Oligoryzomys longicaudatus*). Reservoirs remain persistently-infected, without pathology, despite mounting an immune response that generates high-titer neutralizing antibodies within one to two months after infection [20-23].

The presence of virus-specific IgG antibodies in reservoir rodents infected with their hantaviruses suggests a prominent role for helper T cell (Th)-mediated class switching and affinity maturation. Several Th cell subsets have been identified that provide distinct effector functions that mediate host responses to pathogens [24]. These cells are distinguished by epigenetic expression of specific cytokines, cell surface antigens and transcription factors that results in limited plasticity after differentiation [25]. While these factors have been exhaustively examined in the commonly-used laboratory house mouse (*Mus musculus*), virtually no work has been conducted on natural reservoirs of zoonotic viruses, thus, it is not clear how the coevolutionary adaptations between these viruses and their reservoir hosts have influenced the host responses mediated by helper T cells.

Spillover from natural reservoirs to other rodent species has occasionally been observed in nature. Pinion mice (*Peromyscus truei*) are naturally susceptible to SNV [26,27] but it is unknown if they develop disease, or if they clear the virus or remain persistently infected. Deer mice are experimentally susceptible to ANDV infection without disease; however, unlike SNV infection they clear ANDV [28]. This dichotomous outcome provides an opportunity to study how persistence or clearance occurs in a reservoir host species. The lack of peromyscine-specific reagents has limited the experimental value of deer mice; however, the availability of its genome presents a substantial opportunity for examination of the host response. We devised a computational method to parse the unannotated deer mouse genome for “virtual cDNAs” of interest, which were then used to generate primers for real-time PCR arrays to assess gene expression in lymph node cell cultures from deer mice experimentally infected with Andes virus.

## Results

### PCR array

**Figure 1:**
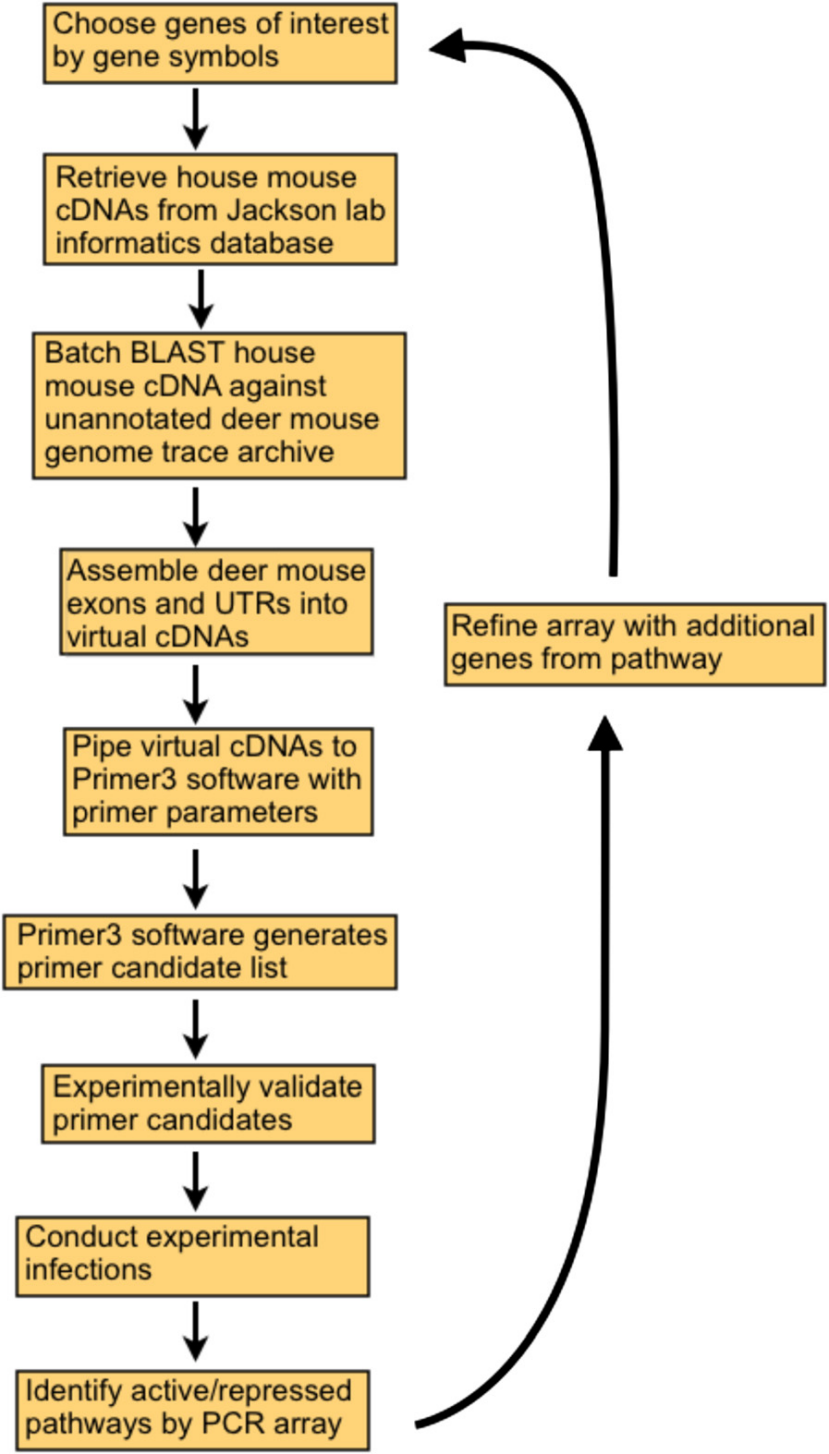
**Algorithm for producing real-time primers for use in deer mouse PCR arrays.** Lab mouse cDNA sequences were BLASTed against the deer mouse genome to produce virtual cDNAs of deer mouse genes. These sequences were then piped to Primer3 software to identify candidate real-time PCR primers. Once pathways of interest have been identified, new genes can subsequently be examined using the same algorithm.

**Figure 2:**
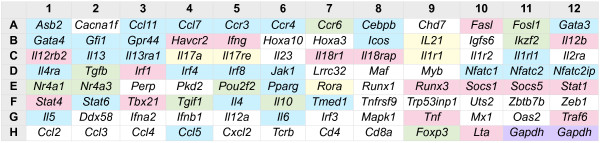
**Deer mouse PCR array.** The array was formatted for a 96-well plate using primers designed by the custom software described in this paper. Wells in pink contain primers that amplify Th1 genes, blue for Th2 genes, green for Treg genes and yellow for Th17 genes. Wells in white amplify various immune genes not involved in Th cell differentiation. Purple wells are *Gapdh*, which is in duplicate and used for sample normalization.

We developed software that uses BLAST of FASTA-formatted cDNA sequences against sequences from the deer mouse genome to identify and assemble exons and untranslated regions into virtual deer mouse cDNAs (Figure 1). We submitted the sequences of 84 house mouse (*Mus musculus*) genes involved in antiviral, innate and adaptive immune responses to the software. The software identified deer mouse orthologs for all of the 84 submitted house mouse immune genes. These deer mouse sequences were then piped to Primer3 software for identification of real-time PCR primer candidates [22,23,29]. Primer candidates for the great majority of deer mouse sequences were identified; however, five genes (*Hopx*, *Id2*, *Il9*, *Il18*, *Cxcl10*) failed to produce candidate primers using our stringent parameters for real-time PCR. *Il9* expression is a marker of Th9 cells, thus its absence prevented us from identifying this phenotype. One primer pair (*Mavs*) produced two products on melt curve analysis and was excluded from the array. The remaining primer pairs amplified single products on melt curve analysis from total RNA collected from concanavalin A-stimulated deer mouse splenocytes and were used with primers from our previous work [22,23,29] to produce a 94-gene PCR array (Figure 2, Additional file 1: Table S1).

### Expression profiles of deer mouse lymph node cell cultures

Fourteen days after inoculation with ANDV, the six deer mice (DM1-DM6) were anesthetized and bled for serology, then euthanized to retrieve cervical lymph nodes, which respond to respiratory infections and are a rich source of antigen presenting cells and virus-specific T cells and B cells. Lymph nodes were not recovered from one deer mouse (DM1), thus no data are presented for this animal. Lymph nodes from the remaining five deer mice were made into single cell suspensions and were plated with or without 10 μg of recombinant ANDV nucleocapsid antigen. Seventy-two hours later, conspicuous lymphocyte proliferation was evident in DM2, DM3, DM4 and DM6 wells with antigen but not in control wells incubated without antigen. Wells containing DM5 lymphocytes failed to proliferate in the presence of antigen and were, thus, excluded from this analysis.

Total RNA was extracted from the cells in each well and cDNA was synthesized using a reverse transcription kit that included a genomic DNA elimination step (RT2 cDNA kit, SA Biosciences). Viral RNA was detected in lymph node cell cultures by reverse-transcription PCR using ANDV S segment-specific primers and ELISA titers of serum samples to ANDV nucleocapsid ranged from 400 to 6400 (Additional file 2: Figure S1); neutralizing antibodies were not examined since they were not expected to be produced until after 21 days post infection [28], a week after the deer mice in this study were euthanized. Sixty-two immune genes were not modulated, whereas 20 genes were upregulated and 12 genes were downregulated. The upregulated genes are involved in Th1/Th2/Treg cell differentiation, class switching and antibody synthesis (notably the IL-4 pathway), inflammation, the type I and III IFN responses and a number of transcription factors and proteins involved in the promotion of cell cycle progression. The array contains 60 genes for the differentiation of helper T cell subsets and 20 of these were modulated. Of the remaining 34 genes involved in other aspects of the host response, including innate and adaptive responses, 12 were modulated.

### Th1 subset markers

**Figure 3:**
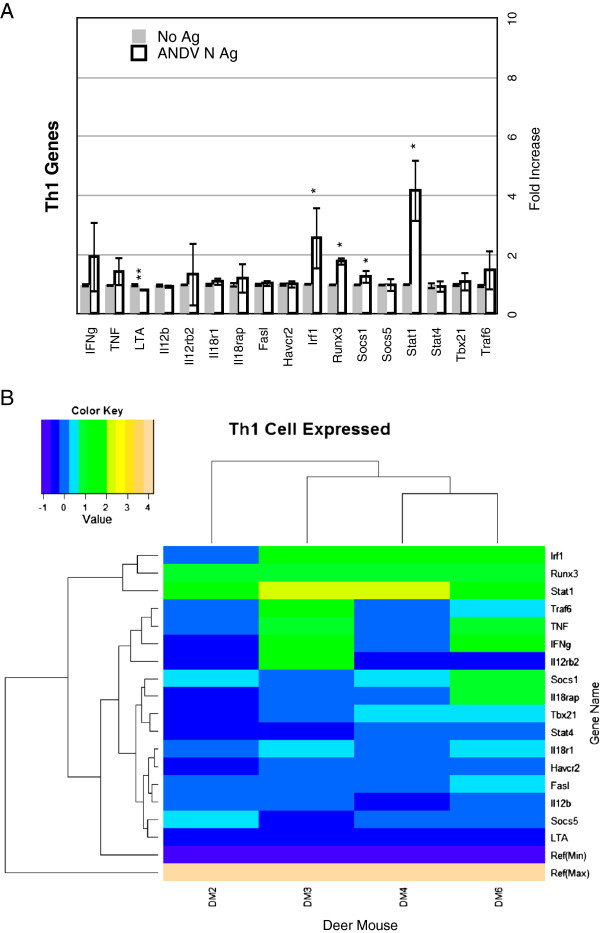
**Th1 gene expression. A.** Bars on the graphs represent the group mean ΔΔCt values. Grey bars are the lymphocyte cultures without antigen and the white bars are the lymphocyte cultures with antigen. The error bars represent the 95% confidence intervals, thus if the error bars do not overlap for a given gene, the gene is significantly upregulated (*) or downregulated (**). **B.** The heat map indicates expression levels of genes from the four deer mice used in this study (DM2, DM3, DM4, DM6). Logfold-changes (“Value”) in the Color Key legend indicate changes in expression. Dendrograms represent deer mouse lymph node cell clusters (top) or gene expression clusters (left).

Variation in samples from lymph node cells incubated without antigen was small for nearly all genes, similar to our observations with uninfected deer mice [22]. For antigen recall experiments, 4 of 17 Th1 genes (*Irf1*, *Runx3*, *Socs1*, *Stat1*) were significantly expressed (24%), whereas one gene (*Lta*) was repressed (Figure 3A). Several genes were substantially upregulated in T cells from some deer mice, but not others (Figure 3B, Additional file 3: Table S2). For example, deer mice DM3 and DM6 expressed 7 Th1 markers (>1.5-fold), whereas DM2 expressed only 2 and DM4 expressed 3. For *Ifng*, a Th1 cytokine, DM3 and DM6 had more than 2.5-fold increased expression, whereas DM2 and DM4 expressed basal levels or less. *Tbx21*, which encodes the T-bet transcription factor, was not upregulated in any of the lymph node cell cultures. T-bet is considered the “master regulator” of Th1 differentiation. Of the two inflammatory cytokines, *Lta* was downregulated and *Tnf* was at basal level. Heirachical clustering grouped DM4 with DM6, and DM3 with DM4/DM6 to the exclusion of DM2, which expressed the fewest Th1 genes. *Irf1*, *Runx3* and *Stat1* expression were grouped as high- expression genes, whereas *Traf6*, *Tnf* and *Ifng* were grouped largely by expression in DM3 and DM6 lymph node cell cultures.

### Th2 subset markers

**Figure 4:**
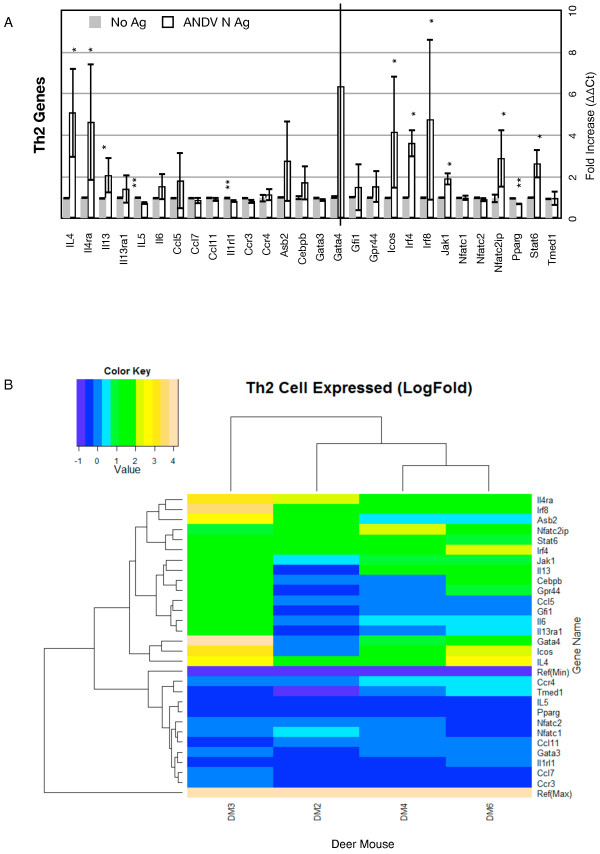
**Th2 gene expression. A.** Bars on the graphs represent the group mean ΔΔCt values. Grey bars are the lymphocyte cultures without antigen and the white bars are the lymphocyte cultures with antigen. The error bars represent the 95% confidence intervals, thus if the error bars do not overlap for a given gene, the gene is significantly upregulated (*) or downregulated (**). **B.** The heat map indicates expression levels of genes from the four deer mice used in this study (DM2, DM3, DM4, DM6). Logfold-changes (“Value”) in the Color Key legend indicate changes in expression. Dendrograms represent deer mouse lymph node cell clusters (top) or gene expression clusters (left).

Of the 28 genes that distinguish Th2 cells, 9 were significantly upregulated (32%) and 3 were downregulated (Figure 4A). Seven of the 10 genes on the array that are part of the IL-4 signaling pathway were upregulated, including genes that are also expressed by activated B cells. *Gata3*, which is the master regulator of Th2 development was unchanged. As with Th1 genes, several Th2 genes were upregulated in some of the deer mice but not others (Figure 4B, Additional file 3: Table S2). DM3 expressed 17 Th2 genes, whereas DM4 and DM6 expressed 12 and 14 Th2 genes, respectively; DM2 only expressed 8 Th2 genes. As with Th1 markers, DM4 and DM6 clustered together; however, DM2 clustered closer to the exclusion of DM3. Gene expression clusters fell into three broad groups of Th2 genes (Figure 4B).

### Treg subset markers

**Figure 5:**
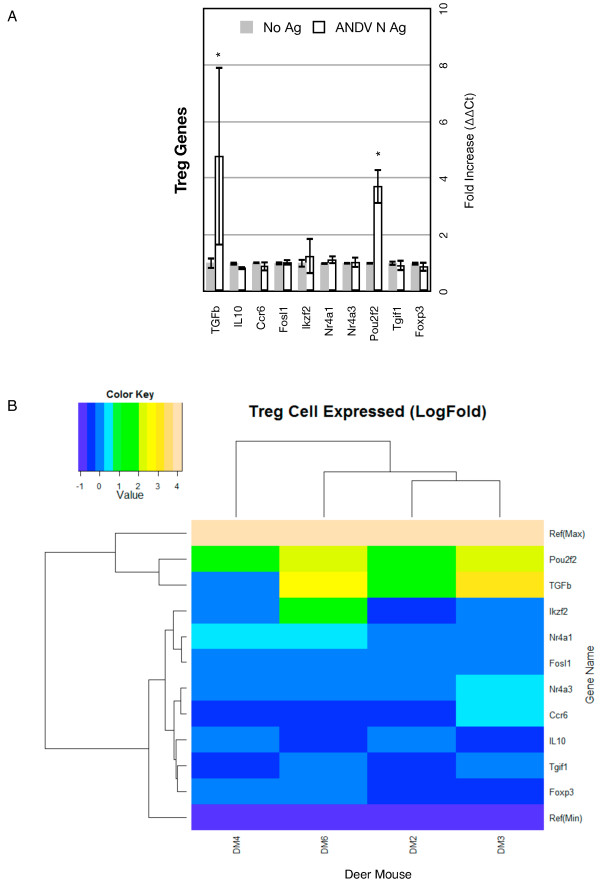
**Treg gene expression. A.** Bars on the graphs represent the group mean ΔΔCt values. Grey bars are the lymphocyte cultures without antigen and the white bars are the lymphocyte cultures with antigen. The error bars represent the 95% confidence intervals, thus if the error bars do not overlap for a given gene, the gene is significantly upregulated (*) or downregulated (**). **B.** The heat map indicates expression levels of genes from the four deer mice used in this study (DM2, DM3, DM4, DM6). Logfold-changes (“Value”) in the Color Key legend indicate changes in expression. Dendrograms represent deer mouse lymph node cell clusters (top) or gene expression clusters (left).

Two markers of Treg cell subsets were upregulated (*Tgfb*, *Pou2f2*) whereas one was slightly repressed (*Il10*) (Figure 5A). *Tgfb* was highly expressed in DM3 (8.43-fold) and DM6 (6.17-fold) and moderately expressed in DM2 (3.62); its expression was near basal levels in DM4. *Pou2f2*, which can be expressed by inducible Tregs, was elevated in all four deer mouse lymph node cell cultures. However, it is also a critical transcription factor in B cells for immunoglobulin synthesis. *Foxp3*, the master regulator of Treg cell development, was not modulated in any of the lymph node cell cultures (Figure 5B, Additional file 3: Table S2). Unlike Th1 and Th2 markers, DM2 and DM3 clustered together, then with DM6 and to the exclusion of DM4. A single gene group clustered with only *Pou2f2* and *Tfgb* expression.

### Th17 subset markers

**Figure 6:**
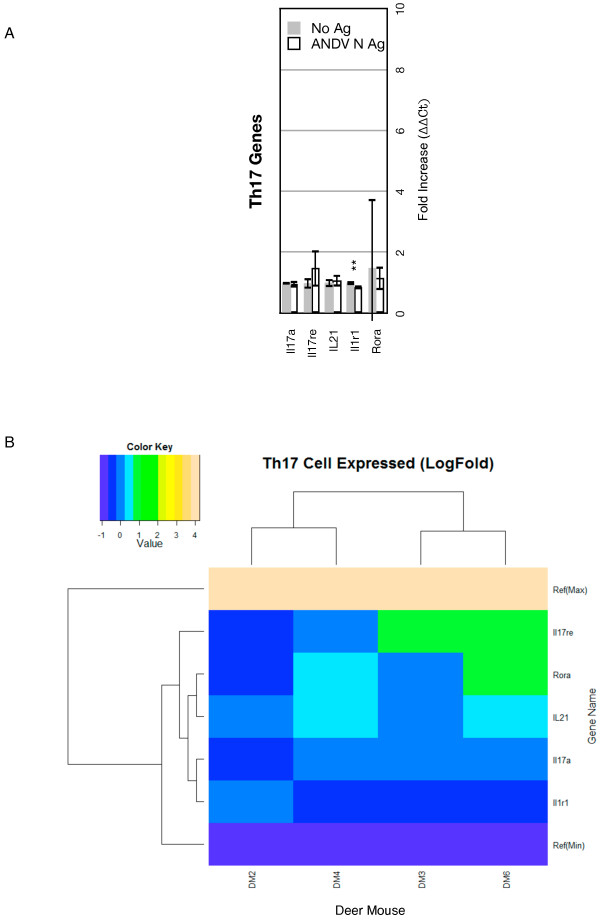
**Th17 gene expression. A.** Bars on the graphs represent the group mean ΔΔCt values. Grey bars are the lymphocyte cultures without antigen and the white bars are the lymphocyte cultures with antigen. The error bars represent the 95% confidence intervals, thus if the error bars do not overlap for a given gene, the gene is significantly upregulated (*) or downregulated (**). **B.** The heat map indicates expression levels of genes from the four deer mice used in this study (DM2, DM3, DM4, DM6). Logfold-changes (“Value”) in the Color Key legend indicate changes in expression. Dendrograms represent deer mouse lymph node cell clusters (top) or gene expression clusters (left).

The *Il1r1* gene, a marker of Th17 cells, was slightly repressed, but none of the five Th17 genes were upregulated (Figure 6A), suggesting this phenotype is not active during the host response to ANDV. However, *Il17re*, a receptor for IL-17C, expression was moderately elevated in DM3 and DM6 (Figure 6B, Additional file 3: Table S2). DM2 and DM4 clustered, and DM3 and DM6 clustered. No clear gene clustering occurred in this group and only two genes were elevated, one in DM3 (*Il17re*) and two in DM6 (*Il17re*, *Rora*).

### Other immune gene markers

**Figure 7:**
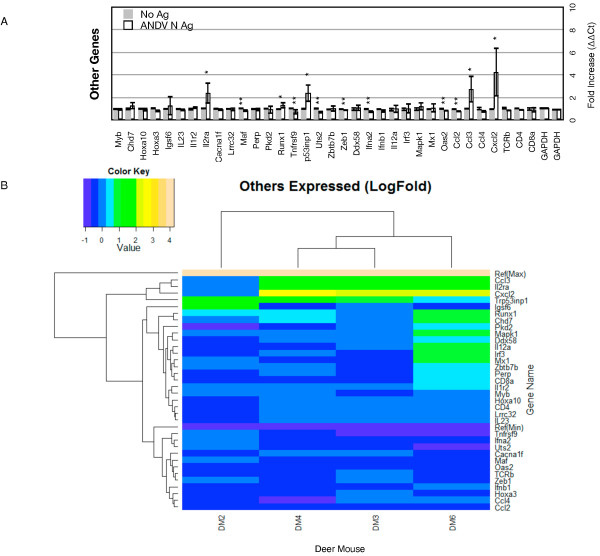
**Other immune gene expression. A.** Bars on the graphs represent the group mean ΔΔCt values. Grey bars are the lymphocyte cultures without antigen and the white bars are the lymphocyte cultures with antigen. The error bars represent the 95% confidence intervals, thus if the error bars do not overlap for a given gene, the gene is significantly upregulated (*) or downregulated (**). **B.** The heat map indicates expression levels of genes from the four deer mice used in this study (DM2, DM3, DM4, DM6). Logfold- changes (“Value”) in the Color Key legend indicate changes in expression. Dendrograms represent deer mouse lymph node cell clusters (top) or gene expression clusters (left).

Of the 34 immune genes not specific for helper T cell subsets, 5 were significantly upregulated (15%) and 7 were downregulated (21%) (Figure 7A). The *Il2ra* gene, which encodes the IL-2Rα chain found on activated T cells and is constitutively expressed by Treg cells, was elevated, as were *Trp53inp1* (cell cycle progression), *Runx1* (Th17 and Treg cells), *Ccl3* (secreted by CTL during antiviral responses) and *Cxcl2* (expressed by macrophages at sites of infection). *Uts2* (vasoconstriction), *Zeb1* (IL-2 gene repression), *Ifna2* (antiviral response), *Maf* (IL-4 expression, susceptibility to apoptosis), *Tnfrsf9* (T cell clonal expansion), *Oas2* (antiviral response) and *Ccl2* (macrophage chemotaxis) were all slightly but significantly downregulated. The remaining genes are involved in various aspects of the immune response, including innate immunity and transition from innate to adaptive immunity; these genes were not modulated in the lymph node cell cultures upon antigen recall. DM3, DM4 and DM6 clustered to the exclusion of DM2 with a single cluster group of four expressed genes (*Ccl3, Il2ra, Cxcl2, Trp53inp1*) (Figure 7B, Additional file 3: Table S2).

## Discussion

The deer mouse is among a growing list of non-model rodent species to have its genome deep sequenced (National Human Genome Research Institute, 2011). Unfortunately, its genome is not annotated and, thus, presents challenges for exploiting it as a research tool. Moreover, considering that more than 50 other species of *Peromyscus* are found in North America and Mexico, its genome is an important resource for other investigators that work with this genus. Thus, we developed a method for rapidly identifying real-time PCR primer candidates for cDNAs of interest for the deer mouse that can be used for quantitative expression analysis. We used this tool to produce a 94-gene immunoarray to examine expression of a variety of T cell differentiation and antiviral genes in lymph node cell recall assays from deer mice infected with ANDV. While transcription data have several limitations, such as an inability to assess translational or post-translational events (e.g., phosphorylation), they can provide important information about how cells respond to antigenic stimuli and what pathways may be candidates for further scrutiny with protein-specific assays. This array showed that several immunological pathways were activated during *in vitro* lymph node cell recall responses to ANDV antigen, with a preponderance of Th2-like gene expression.

The majority of expressed subset markers were suggestive of Th2 cells (9 of 15 genes, 60%). Th2 cells historically have been considered important for antibody responses to extracellular pathogens and toxins and less important for viral infections. However, most studies examining T cell responses have focused on pathogenic viruses and since ANDV is not pathogenic in deer mice it may be that such a response is protective from persistent infection. Th1 and Treg genes were also upregulated, but no indication of a Th17 response, suggesting a mixed Th1, Th2 and Treg cell response. We found similar results with primary T cell lines from deer mice at 10 days post infection with SNV, in which *Ifng*, *Il4*, *Il5*, *Il10* and *Tgfb* were elevated [23]. However, with ANDV infection, neither *Il5* or *Il10* expression appear elevated. It is noteworthy that the T cell lines from SNV infected deer mice in this previous work were of spleen origin, collected at 10 dpi and propagated on bone-marrow derived APC that were likely dendritic cells. In the present work, lymphocytes from lymph nodes were used, thus it is possible that cell source, timing and culturing methods could alter the expression of genes. However, since the present work used freshly isolated lymph node cells it is likely to be more relevant to natural infection compared to T cell lines, which require months to generate, may select for rare clones and which may be influenced by developmental cues from the APCs. Examination of IL-5, which is closely linked to IL-4 in other mammalian species and likely so in deer mice, and IL-10 may be important in the different outcomes of deer mice infected with ANDV (clearance) or SNV (persistence).

Seven of the 10 genes of the IL-4 signaling pathway on the array were upregulated; *Il4*, *Il4ra*, *Jak1*, *Stat6*, *Nfatc2ip*, *Il13* and *Irf4*. *Socs1*, *Gata3* and *Il4rc*, also part of the pathway, were not modulated. Activation of Th2 cells results in the dimerization of Nfatc2ip with IRF4, which were in the same expression cluster group (Figure 4B), within those cells and which is a transactivator of the IL-4 promoter [30]. IL-4 is then secreted from Th2 cells and binds to its receptors on target cells, including B cells, where it activates the Jak1 pathway and induces dimerization of IRF4/STAT6 (which also clustered with *Irf4* and *Nfatc2ip*) that leads to B cell activation, germinal center formation, class switching, maturation to plasma cells, affinity maturation and antibody secretion [31,32]. IL-4-stimulated B cells that also have CD86 (B7-2) engaged (from T cell CD28) increase expression of *Pou2f2* which, in laboratory house mice, leads to increased synthesis and secretion of IgG [33]. *Pou2f2* is also a marker of Treg cells, thus we cannot discriminate its expression in lymph node cultures, which have T cells and B cells. Considering the number of genes in this pathway that are expressed during ANDV infection, additional genes of the pathway should be examined. The IL-4 intercellular communication pathway has more than 20 proteins involved in cognate interactions of T cells and B cells, thus expression analysis and examination of phosphorylation events should be a high priority for future work.

Interferon-γ (IFNγ) induces the expression of several transcription factors, including members of the interferon response factor (IRF) family [34]. IFNγ ligation induces the activation of STAT1, expression of which was elevated in all of the deer mouse lymph node cell cultures (Figure 3A, Additional file 3: Table S2), that, in turn, activates a number of other factors, including IRF1. IRF1 directs the differentiation of antigen-stimulated primary T cells towards the Th1 phenotype. IRF4 controls a variety of maturation events in both T and B cells, including the maturation of T cells toward the Th2, Th9 or Th17 phenotypes [35], and induction of class switching, which precedes somatic hypermutation leading to affinity maturation [36], and plasma cell formation in B cells, which also requires IL-4 and IL-6 [37]. IL-21, which contributes to germinal center plasma cell formation in the laboratory house mouse, was not upregulated. It is also involved in expression of MHC class II molecules in dendritic cells that are critical for initiating helper T cell responses to antigens. IRF4 expression in follicular helper T (Tfh) cells is essential for germinal center formation [35]. IRF8 is expressed by germinal center B cells, but not plasma cells, where it participates in the regulation of more than 50 genes involved in B cell activation and maturation, and interaction with Tfh cells and follicular dendritic cells that present antigen in its native confirmation to germinal center B cells [38]. *Runx3* is expressed by B cells and promotes proliferation, presumably during clonal expansion induced by Th1 cells [39].

Both deer mice that had increased levels of *Ifng* mRNA (DM3 and DM6) also had high levels of *Tnf* mRNA; however, infection with ANDV does not cause conspicuous inflammation in deer mice [28]. Considering that IFNγ induces activation of STAT1, which was significantly elevated in all four deer mice, it is unclear how its absence results in increased *Stat1* gene expression; however, by 3 days post culture it is possible that *Ifng* expression had already subsided in the two cultures that were *Ifng*-negative. In addition to its role in T cell activation, STAT1 is also the major signaler/transcription factor for type I and type III IFNs and is typically a marker for innate signaling. In the LN cultures there is probably little or no active virus replication (although viral RNA was detected), but the innate response might be triggered by addition of antigen. It is also noteworthy that the important regulators of Th1, Th2 and Treg cells (T-bet, GATA3, Fox-p3, respectively) for the laboratory house mouse are not upregulated in deer mice infected with ANDV, yet many genes associated with the expression of these regulators are expressed, nonetheless. It may be that expression of those genes is unnecessary (i.e., the proteins are already present), or that by 14 days post infection they are no longer needed and are subsequently repressed. Most transcription factors are tightly regulated because they have enzyme-like activity; small amounts can profoundly affect downstream signaling cascades and gene expression. Regardless, it is clear that genes associated with three helper T cell subsets are expressed during the immune response to ANDV. It will be necessary to examine the activities of these transcription factors to clarify their roles; fortunately, many of these proteins are highly conserved and antibodies to their orthologs in other species (e.g., laboratory house mouse) are likely cross-reactive with many deer mouse proteins. The small sample size presented here (4 deer mice) limits interpretation of these data; however, the number of genes examined should allow extensive examination of the host response to clarify the pathways involved in deer mouse responses to infectious agents.

The development of software that allows rapid deployment of real-time PCR expression arrays for the deer mouse should help clarify the immunological events that govern hantavirus persistence or clearance from rodent reservoirs. The data presented here demonstrates that such work can be done, and that it generates substantial amounts of data indicating which pathways may, or may not, be involved in the host response. This should permit more efficient examination of these pathways using commercially available, cross-reactive antibodies to specific signaling molecules that may play decisive roles in hantavirus clearance.

## Conclusions

We developed a rapid method for designing real-time PCR arrays for the deer mouse that can be used to study the host immune response to viral infections. Lymph node T cells from deer mice infected with ANDV express mostly genes of the Th2 phenotype but fewer genes of Th1 and Treg phenotypes. Seven genes of the IL-4 signaling pathway were elevated in the array, suggesting this cytokine may play a role in clearance of ANDV from deer mice.

## Methods

*Ethics statement and infections*. All procedures using deer mice were in compliance with the USA Animal Welfare Act and approved by the Rocky Mountain Laboratories institutional animal care and use committee and performed following the guidelines of the Association for Assessment and Accreditation of Laboratory Animal Care, International (AAALAC) by certified staff in an AAALAC-approved facility. All work involving infectious materials was performed in the biosafety level-4 laboratory at RML. Six deer mice were intramuscularly-infected with 200 FFU ANDV-9717869 in the hind limbs. Fourteen days later, deer mice were anesthetized by respiratory delivery of isoflurane for cardiac blood collection, then euthanized by thoracotomy. Lymph nodes were not recovered from one deer mouse (DM1) and another deer mouse’s lymphocytes (DM5) failed to proliferate *in vitro* in response to antigen. These two animals were excluded from the analysis in this work. Thus, the data were from the four remaining deer mice (DM2, DM3, DM4, DM6).

*Lymphocyte recall assay*. Cervical lymph nodes were collected in PBS and made into single cell suspensions in serum-free Hank’s balanced salt solution (HBSS). Cells were washed twice in HBSS, then once in complete medium (CM, 5% FBS RPMI-1640) and adjusted to 10^7^/ml in CM. For each deer mouse, 500 μl of cells (5x10^6^) were pipetted into wells of a 24-well plate in duplicate; one well received 500 μl of CM (basal gene expression levels) and the other received 500 μl of recombinant ANDV nucleocapsid (N) antigen in CM for a final concentration of 10 μg/ml. Cells were incubated for 72 hours at 37°C and 5% CO_2_ then collected and treated with RLT buffer for RNA extraction (RNA Easy kit, Qiagen). Following inactivation (according to approved standard operating procedures) the material was transferred from the BSL-4 to BSL-2 and frozen at −70°C.

*Genome mining and primer design*. Eighty-four genes of immunologic and antiviral relevance were selected to expand the deer mouse array previously describe by us [22]. Laboratory house house mouse (*M. musculus*) cDNA sequences were downloaded from Jackson Laboratories Informatics (http://www.informatics.jax.org/) and BLASTed against the unannotated deer mouse genome (ftp://ftp.ncbi.nih.gov/pub/TraceDB/peromyscus_maniculatus/) to generate cDNAs using software developed by us (http://dna.publichealth.uga.edu/BlastPrimer/BlastPrimer.php). A stringent threshold cutoff of 1e^-10^ was used in this software to minimize erroneous hits in the database. The BLAST results were then aligned and merged into “virtual cDNAs”, then compared to the house mouse sequence. Gaps in the peromyscus sequences were filled with ‘N’ to maintain approximate transcript size based upon the laboratory house mouse transcript length. Where nucleotide differences occurred, which could have been polymorphisms or sequencing errors, the most common nucleotide was used (“majority rule”). The virtual cDNAs were piped to Primer3 software (http://frodo.wi.mit.edu/) to generate candidate primer lists and product size estimates (product size 75–120 bp, percent G +C 47–53%) as previously described [29,40]. Candidate primers were selected and validated for SYBR green PCR using deer mouse splenocytes stimulated with 2 μg/ml concanavalin A (C-0412, Sigma, St. Louis, MO) in CM overnight prior to RNA extraction and real-time PCR of single products as described in the next section.

*PCR array*. Primers (Additional file 1: Table S1) were purchased in two 96-well plates, with forward primers in one and reverse primers in the other (Operon, Huntsville, AL). Primers were resuspended to 100 μM in water and combined in a third 96-deep well plate (Axygen P-1ML-SQ-C) to 10 μM (500 μl per well final volume) that was sealed with a silicone mat (Axygen CM-96-RD). Two μg of total RNA were reverse transcribed into cDNA using the RT2 cDNA Synthesis Kit (SABiosciences), which contains a genomic DNA elimination step. The cDNA was added to RT2 SYBR green I master mix (SABiosciences) and mixed thoroughly. Twenty microliters were dispensed into a 96-well real-time PCR plate using an 8 channel pipettor, then 5 μl of primers (2 μM final concentration) of primers were added. Cycling was 95°C for 30 sec and 60°C for 30 sec for 40 cycles, followed by an 80-step melt curve analysis (Bio-Rad iQ5 thermal cycler). The ΔΔCt method [41] was employed using the mean of *Gapdh* as the reference within samples for normalization, and comparison of normalized samples between antigen-stimulated and CM only to calculate fold change. Heat maps and hierarchical clusters were generated using the heatmap.2 (“gplots” package) from R statistical software (http://www.r-project.org/). Distance matrix was calculated using the Euclidean method, and a complete linkage clustering method was used for generating the hierarchical clusters. To allow identical color codes across cell gene sets, maximum and minimum logfold changes across all gene expression were included as references indicated by Ref(Max) and Ref(Min).

## Abbreviations

HCPS: Hantavirus cardiopulmonary syndrome; SNV: Sin Nombre virus; ANDV: Andes virus.

## Competing interests

The authors declare that they have no competing interests.

## Authors’ contributions

TS, HF and JP designed experimental protocols and executed laboratory work. TIS and TG developed the software for mining the deer mouse genome, parsing Primer3 and statistical analysis of gene expression levels. TS wrote the manuscript. All authors read and approved the final manuscript.

## Supplementary Material

Additional file 1: Table S1 Primers for the real-time PCR array, listed 5^′^ to 3^′^. Click here for file

Additional file 2: Figure S1 Detection of ANDV genome by reverse transcription PCR and antibody titers from deer mice used in this work. Primers specific for the ANDV S segment were used to amplify product from cDNA of antigen-stimulated lymph node cell cultures. Antibody endpoint titers to ANDV nucleocapsid antigen are presented below the gel and are the reciprocals of the greatest dilutions that were 0.200 OD above the mean of the negative control wells. Click here for file

Additional file 3: Table S2 Gene expression levels in T cell recall to ANDV nucleocapsid antigen. Cells in pink are 1.5-fold or more increased. No gene was decreased 50% or more. The numerals in red font indicate the number of genes in that category that were elevated for each deer mouse. Click here for file
