# Supplementary material for: Expression profiling of lymph node cells from deer mice infected with Andes virus
Source: BMC Immunol. 2013 Apr 9;14:18. doi: 10.1186/1471-2172-14-18 (PMC3637227; doi:10.1186/1471-2172-14-18)
Supplement: Additional file 1: Table S1 — Primers for the real-time PCR array, listed 5′ to 3′. [file 1471-2172-14-18-S1.pdf]

Table S1. Primers for the real-time PCR array, listed 5' to 3'.

Gene	Forward	Reverse
Asb2	GTGCATCAGCCCTTTATGAGG	CATCCTTGTTGGCTTTGTTGG
Cacna1f	TGCAGTGTGTGTTTGTGGC	ACCAATGCAGGCGAACAT
Ccl11	TCTCACTGTGTAACCCTGGCT	GCAGGTGGATCTCTGTGAGTT
Ccl7	TCTGCCAGCTCTCACTGAA	AGCAGCAGGCACAGAAGTATG
Ccr3	TTGGCAATTTCTGACCTGC	ATGTGATGGCCAAAACGC
Ccr4	GCAGGTGGATCTCAGTATGTG	CTCTGTATGTAACAGCCCTGG
Ccr6	TGCTCCTTGGAAGAGGTCA	CCACCATAATATTGCCCAGG
Cebpb	TGCTCCTTGGAAGAGGTCA	GCAAAGGTCATCACCACCA
Chd7	ATGCAGCAGAAGTTGGTGG	GCTGCTTGAACAAGTTCCG
Fasl	ACTTCCGGGGTCATTCTTG	TCCATTAGCACCAGTTCCC
Fosl1	TCCTCCTGAATGGCACAAC	CTTGATGTGGAGGCTTGGA
Gata3	AGTCCGCATCTCTTCACCTTCC	GGCACTCTTTCTCATCTTGCCG
Gata4	AGGGTGAACCTGTGTGCAA	TGGGCTTTCGTTTTCTGG
Gfi1	GCATCAAATGCAGCAAGG	ATCTCGCACGCAAATGGT
Gpr44	TGCAAGTTTCACTCCTCGG	TGGTTTTGTGCCCACACT
Havcr2	GAATGCATATCTGCCCTGC	CCAATTGGCACACCATGA
IFNg	GGCTATTTCTGGCTGTTACTGCC	ATCCCCGACATCTGAGCTACTTG
Hoxa10	AAAGCACCAAACGCTGGA	CCAAATCTTGACCTGCCTG
Hoxa3	AGCGTTTGAAAACCGTGG	CAGAATGTGAGCAGCAAGGA
Icos	GGTGTGCAGCTTTTATTGTGG	TCACTATTGGGGTCATGCG
IL21	AACTCAAGCCAGCAAACACAGG	GCTGCTTTTTCTCAGCCTTGG
Igsf6	AGAAGCCTGTGGTTTCGCT	TTGGTTCTGGTCCAGGGTT
Ikzf2	TTTCTGCCATGCATCCCT	TTCCTGCCTTCCATCACAA
Il12b	TGTTCTCATGGGCTGATCC	GGCAGCCTTGGTTGAAAA
Il12rb2	CCTCCGTGGGACATCATAA	TGAGTCGATTGCGAACCA
Il13	TGCAAACCCATCTACAAGACCC	GCCACTTCGATTTTGGTATCCG
Il13ra1	TGGTGTTCTTCCTGATGCTG	AGCGCTTCGCTCCAATTA
Il17a	AGTATCCCTCTGTGATCTGGGAGG	GGATCTCTTGCTGGATGAGAACAG
Il17re	TCCTTTGATTTGCTGCCC	TTCACATTCCAGTGCCCA
IL23	AGAAATGATGTCCCCCGTATCC	CAGACCTTGGTGGATTCTTTGC
Il18r1	TAACCAGACCAGGCTCGAA	AAGTGCAGCTGTTGGTTGG
Il18rap	AACACAACACGGACCATCC	CTGCGACCAAAATGCTCA
Il1r1	TGCAAAGCCAGGTTCACA	TTTGTGCTCACCCATAGCAG
Il1r2	TATGTGGAAGTGGCCCTGA	TGGATTCCTGGCAACACA
Il1rl1	AATGCAGGGGACCATCAA	TTGGCACTGGCATTTGG
Il2ra	TGCCACATTCAAAGCCCT	CCAACTCCTTTGTTCTTCGG
Il4ra	AGAACCCTGTTCCCAACCA	TTGGATGGCAACTCCATGT
TGFb	CGTGGAACTCTACCAGAAATACAGC	TCAAAAGACAACCACTCAGGCG
Irf1	TCCCAAAGAATTGCTGCC	CCACACAGGACCCCATTTT
Irf4	CAGATGGGCTCTATGCCAA	TTTGTTGGGCCGATCACT
Irf8	AGGTGACCCGGAAGTTGTT	GCCGTTTCACAAACACACC
Jak1	TCCCTTCGAATGACAGTGG	ACATTTGGTTTCTGCCGC
Lrrc32	ATTGGAACTGGGCACCAA	CCAAGCTGGCAAAGGTGTA
Maf	TTATTCGCCCCTTGTTGC	TGGGTGCAAGTTTGTCCA
Myb	AGGCACAGCATTGTTCCAC	TTGGGAGTTGAGGTGCTGA
Nfatc1	CTGCCCACTGGATTCAAAC	TGCTTGCTTCCACAATGC

Gene	Forward	Reverse
Nfatc2	TTAAGCCGCATGCCTTCT	TTGGTGTTGCCCACAATCT
Nfatc2ip	TCCGGGGATCTCATTGAA	TAGCCCTTATGGGGCAAA
Nr4a1	AAGCCTTTCAGCCTCCAAC	ATCGGAACCTGCCATTCA
Nr4a3	TGGCACGCTCATTTTCCT	TTCCATGAAGAACGGCCT
Perp	CTCTTCTGCGGCTTCATCA	AAAACGAGCATCTGGGGTC
Pkd2	ATTTGGACCGCGAAACG	TGGCAATGATTCCCCAGT
Pou2f2	CCAAAATGCTTGGAACCG	AAGGTGCGAGCGAATTGT
Pparg	TTTCCGAAGAACCATCCG	GGCACTTCTGGAAACGACA
Rora	TGCAACAGTGGCAACCAT	CACAAAAGGAGCAGGCAGA
Runx1	GGGCGAATCACACTGAATG	TCCTTTCGAAAACGCACC
Runx3	CCAGGCTACAGCAAGAAACA	TCCCCACATCCTCTGTTCA
Socs1	TGAGATCGCGAAGAACCTG	GGAAGGGGAAGGAACTTCA
Socs5	CGGTTTGGGGACCATTTTA	TCAATCCCCGTTCTGCAT
Stat1	GGTGCATCATGGGCTTTA	TGAATGTGATGGCCCCTT
Stat4	AACCATTTACCTTCTGGACCTGG	TTGCTCACGAAGCCCATGATGTACC
Stat6	CGCTTTAGCGACTCTGAGATTGG	TCTTTGGCAGAAAATGGCTGG
Tbx21	GCCAACCCCAAGGATATGGT	GAATGTGGGCTTCATGCTC
Tgif1	AAACGGGCAGCAAAGATG	CTCTTGCCATCACCCTTGA
Il4	CCCCGTGCTTGAAGAACAATTC	GGACTCATTCCCAGTACAGCTTTTC
Il10	TAAGGGTTACCTGGGTTGCCAAG	CAAATGCTCCTTGATTTCTGGGC
Tmed1	CGGAGCATCCAGATGATGA	ACATTGGCAGCTGACCCTA
Tnfrsf9	ATAGATGGTTGTGAGCCACC	TGTTAAGAGCACTGGCTGC
Trp53inp1	ATGAGCCAACCACACTTGC	GGGAAATGCGCTCTTTGA
Uts2	GGCTTTCTCTGGACAAGACTC	TCCAGAAGCATTCCGAGAC
Zbtb7b	TGCCCAATGGTGAAGACA	AAACGCTTTTCGGGGCT
Zeb1	CCCAACAAACAGCTGCAA	ATCCACAGCCCCATCAAA
IL5	GAAGAATCAAACTGTCCGTGGG	ACACTGCTCTTTTTGGCGGTC
Ddx58	CCAATGGAAAGGTTGGGA	TGGGTTTGATTCTGGCCT
Ifna2	ACTCATCTGCTGCTTGGGA	TTGCAGGTCTTTGAGCTGG
Ifnb1	TGCCTTCGTCATCCAAGAG	TCTCATTCCACCCAGTGCT
Il12a	TCCAAAAACCTGCTGAGGACCAC	AATCAACGTCTTCAGGAGTGCAG
Il6	CCATCCAACTCATCCTGAAAGC	CCACAGATTGGTACACATAGGCAC
Irf3	TAGAAAAGGAAGCCCCAGC	TGGTGCCAACACTGGTTTC
Mapk1	TGCTGTGGAGTTGATGGTG	AACAGTCATGCAAGGGCA
Tnf	TGTAGCCCACGTTGTAGCAAACC	CTGGTTGTCTTTGAGATCCATGC
Mx1	ATTGCTGGGGAAAAACTGG	TGATGCCAGGCAGATCAA
Oas2	TGGACGAGTTCGACATTGC	AAGATGCAGAGCTGCTGGT
Traf6	TTGTCCCCAATGTCAACG	AATGGCATGGACACAGCA
Ccl2	CAGACGTACACAAGAAAACTGGACC	GTCAAGTTCGCATTCAAAGGTGC
Ccl3	AGCCAGGTGTCATTTTCCTAACC	CAGCTCAGTGATGTATTCTTGGACC
Ccl4	TGCTGCTTTTCTTACACTGCTCG	TCTAGTGAGGAATACCACTGCTGGC
Ccl5	CCACGTCAAGGAGTATTTCTACACC	TCCTGAACCCACTTCTTCTTTGG
Cxcl2	ATGGTCAGGAAGTTTGCCTCG	TACAGCAGCCCATACTCCGATTC
TCRb	CAATAACATCACCTACTGCCTGAGC	GTAAGCCCATAGAACTGGACTTGG
CD4	GGTTGAAATGAAGGACCCTGAGG	CCTCTGGATGAAACCTGGATTTTGG
CD8a	AAGAAGAGCGGATTGGACTTCG	AGATGAGAGTGATGACCAGGGACAG
Foxp3	AAGCAGATCACCTCCTGGATGAG	TAGCACCCAGCTTCTCCTTTTCC

Gene	Forward	Reverse
Lta	ATGGTGTCTCCCATCTACACTTCAG	TTGAAACGGTCAGCATGGAGG
GAPDH	GGTGCCAAAAGGGTCATCATCTC	GCAGGAAGCGTTGCTGATAATCTTG
