# Supplementary figures and images for: Expression profiling of lymph node cells from deer mice infected with Andes virus
Source: BMC Immunol. 2013 Apr 9;14:18. doi: 10.1186/1471-2172-14-18 (PMC3637227; doi:10.1186/1471-2172-14-18)

**Figure S1.**

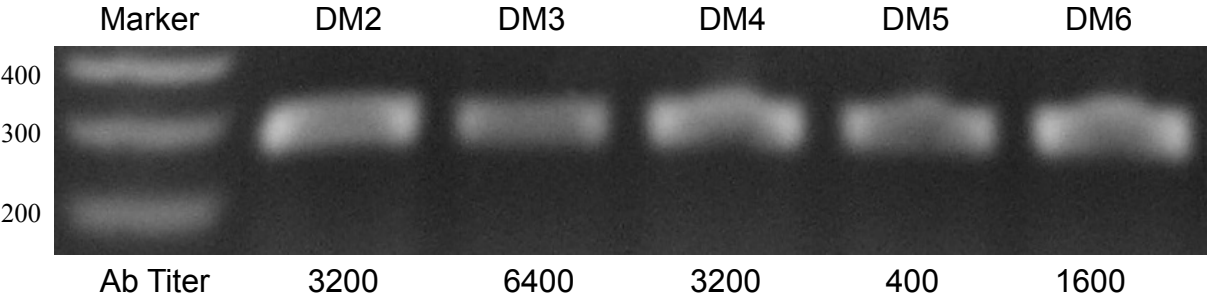

Supplement: Additional file 2: Figure S1 — Detection of ANDV genome by reverse transcription PCR and antibody titers from deer mice used in this work. Primers specific for the ANDV S segment were used to amplify product from cDNA of antigen-stimulated lymph node cell cultures. Antibody endpoint titers to ANDV nucleocapsid antigen are presented below the gel and are the reciprocals of the greatest dilutions that were 0.200 OD above the mean of the negative control wells. [file 1471-2172-14-18-S2.pdf]
