# Supplementary material for: Expression profiling of lymph node cells from deer mice infected with Andes virus
Source: BMC Immunol. 2013 Apr 9;14:18. doi: 10.1186/1471-2172-14-18 (PMC3637227; doi:10.1186/1471-2172-14-18)
Supplement: Additional file 3: Table S2 — Gene expression levels in T cell recall to ANDV nucleocapsid antigen. Cells in pink are 1.5-fold or more increased. No gene was decreased 50% or more. The numerals in red font indicate the number of genes in that category that were elevated for each deer mouse. [file 1471-2172-14-18-S3.pdf]

Table S2. Gene expression levels in T cell recall to ANDV nucleocapsid antigen. Cells in pink are 1.5-fold or more increased. No gene was decreased 50% or more. The numerals in red font indicate the number of genes in that category that were elevated for each deer mouse.

Gene	DM2	DM3	DM4	DM6
**Th1 expressed**	2	7	3	7
**IFNg**	0.77	3.13	0.98	2.88
**TNF**	1.08	1.98	0.94	1.74
**LTA**	0.84	0.81	0.77	0.84
**Il12b**	0.98	1.00	0.82	0.94
**Il12rb2**	0.76	3.02	0.80	0.80
**Il18r1**	0.87	1.23	1.13	1.20
**Il18rap**	0.74	1.14	0.99	1.98
**Fasl**	1.02	1.08	0.88	1.21
**Havcr2**	0.79	1.14	1.00	1.14
**Irf1**	1.11	3.22	3.57	2.42
**Runx3**	1.61	1.71	1.87	1.97
**Socs1**	1.23	0.98	1.27	1.61
**Socs5**	1.35	0.81	0.90	0.89
**Stat1**	2.96	5.01	5.19	3.57
**Stat4**	0.66	0.86	1.14	1.05
**Tbx21**	0.67	1.00	1.25	1.47
**Traf6**	1.00	2.51	1.00	1.48
**Th2 expressed**	8	17	12	14
**IL4**	2.61	6.75	3.88	7.19
**Il4ra**	5.26	8.49	2.44	2.40
**Il13**	0.77	2.44	2.45	2.69
**Il13ra1**	0.86	2.45	1.02	1.38
**IL5**	0.81	0.83	0.68	0.67
**Il6**	1.04	2.54	1.31	1.33
**Ccl5**	1.15	3.93	1.07	1.16
**Ccl7**	0.77	1.18	0.76	0.86
**Ccl11**	1.08	0.80	0.92	0.90
**Il1rl1**	0.81	0.81	0.79	0.98
**Ccr3**	0.71	1.02	0.83	0.82
**Ccr4**	0.94	0.89	1.22	1.60
**Asb2**	2.51	5.72	1.56	1.34
**Cebpb**	0.88	2.56	1.09	2.44
**Gata3**	0.77	0.96	0.91	0.98
**Gata4**	0.89	18.83	2.15	3.57
**Gfi1**	0.77	3.28	1.11	0.91
**Gpr44**	0.81	2.54	0.98	1.87
**Icos**	0.90	7.70	3.99	4.10
**Irf4**	3.00	3.08	4.04	4.42
**Irf8**	2.34	10.74	2.92	3.11
**Jak1**	1.56	2.39	1.91	1.86
**Nfatc1**	1.19	0.98	0.99	0.84
**Nfatc2**	0.97	1.04	0.98	0.73

Gene	DM2	DM3	DM4	DM6
Nfatc2ip	2.45	1.85	5.05	2.29
Pparg	0.83	0.67	0.70	0.69
Stat6	2.35	2.58	3.69	1.99
Tmed1	0.63	0.83	0.96	1.51
Treg expressed	2	2	1	3
TGFb	3.62	8.43	0.93	6.17
IL10	0.92	0.71	0.87	0.84
Ccr6	0.82	1.19	0.80	0.77
Fosl1	0.94	0.92	1.05	1.17
Ikzf2	0.83	0.96	0.98	2.23
Nr4a1	0.92	1.05	1.26	1.27
Nr4a3	0.94	1.34	0.93	0.91
Pou2f2	3.28	4.16	3.06	4.36
Tgif1	0.79	1.00	0.71	1.18
Foxp3	0.64	0.82	1.02	1.08
Th17 expressed	0	1	0	2
Il17a	0.85	1.09	0.87	1.02
Il17re	0.83	2.01	1.07	2.02
IL21	0.89	0.91	1.22	1.28
Il1r1	0.93	0.77	0.85	0.86
Rora	0.71	1.04	1.19	1.69
Others expressed	2	4	4	10
Myb	0.93	0.76	1.06	1.07
Chd7	1.05	0.94	1.45	1.65
Hoxa10	0.67	1.11	1.06	0.96
Hoxa3	0.81	1.06	0.71	0.76
Igsf6	2.59	1.06	0.69	0.77
IL23	0.70	0.95	0.96	1.05
Il1r2	1.00	1.00	1.13	1.26
Il2ra	0.95	2.92	3.08	2.67
Cacna1f	0.86	1.05	1.10	0.78
Lrrc32	0.74	0.96	0.92	1.17
Maf	1.02	0.84	0.82	0.69
Perp	0.82	0.84	0.90	1.24
Pkd2	0.47	1.07	0.82	1.46
Runx1	1.29	0.98	1.31	1.75
Tnfrsf9	1.15	0.59	0.64	0.59
Trp53inp1	2.56	2.14	3.45	1.48
Uts2	0.93	0.74	0.67	0.56
Zbtb7b	0.94	0.76	0.94	1.47
Zeb1	0.91	0.89	0.83	0.85
Ddx58	0.78	1.12	0.98	1.52
Ifna2	0.98	0.80	0.64	0.67
Ifnb1	0.65	0.83	0.71	1.11
Il12a	0.73	0.96	0.78	1.63
Irf3	0.64	0.82	0.94	1.70
Mapk1	0.94	1.00	1.14	1.77

Gene	DM2	DM3	DM4	DM6
**Mx1**	0.89	0.86	0.86	1.71
**Oas2**	0.82	0.77	0.86	0.83
**Ccl2**	0.77	0.83	0.65	0.83
**Ccl3**	1.00	3.35	2.86	3.75
**Ccl4**	0.76	0.96	0.59	0.90
**Cxcl2**	0.96	4.98	5.76	5.33
**TCRb**	0.84	0.98	0.84	0.83
**CD4**	0.77	1.02	0.91	0.99
**CD8a**	0.84	0.71	0.80	1.30
**GAPDH**				
**Replicate 1**	0.90	1.18	1.11	1.08
**Replicate 2**	1.11	0.85	0.90	0.92
